# Vascular endothelial growth factor gene polymorphisms and the risk of renal cell carcinoma: Evidence from eight case-control studies

**DOI:** 10.18632/oncotarget.14263

**Published:** 2016-12-27

**Authors:** Mancheng Gong, Wenjing Dong, Zhirong Shi, Shaopeng Qiu, Runqiang Yuan

**Affiliations:** ^1^ Department of Urology, Zhongshan Affiliated Hospital of Sun Yat-sen University, Zhongshan, Guangdong 528403, China; ^2^ Department of Oncology, Zhongshan Affiliated Hospital of Sun Yat-sen University, Zhongshan, Guangdong 528403, China; ^3^ Department of Pharmacy, The Second People's Hospital of Zhuhai, Zhuhai, Guangdong 519020, China; ^4^ Department of Urology, The First Affiliated Hospital of Sun Yat-Sen University, Guangzhou, Guangdong 510080, China

**Keywords:** vascular endothelial growth factor, VEGF, renal cell carcinoma, gene polymorphism, meta-analysis

## Abstract

**Background:**

Vascular endothelial growth factor (VEGF) protein plays important role in renal cell carcinoma (RCC) development and progression. *VEGF* gene polymorphisms can alter the protein concentrations and might be associated with renal cell carcinoma risk. However, the results of studies investigating the association between *VEGF* polymorphisms and renal cell carcinoma risk are inconsistent. Thus, a meta-analysis was performed.

**Methods:**

We selected eligible studies via electronic searches. Only high-quality studies were included based on specific inclusion criteria and the Newcastle-Ottawa Scale (NOS).

**Results:**

Eight studies primarily focusing on seven polymorphisms were included in our meta-analysis. Our results showed dramatically high risks for renal cell carcinoma were found regarding most genetic models and alleles of the +936C/T polymorphism (except CT vs. CC). In addition, significant increased renal cell carcinoma risks were found regarding all genetic models and alleles of the -2578C/A polymorphism. However, no significant associations were found between renal cell carcinoma risk and the +1612G/A, -460T/C, -634G/C, -405G/C or -1154G/A polymorphisms.

**Conclusions:**

Our meta-analysis indicates that the +936C/T and -2578C/A polymorphisms of *VEGF* are associated with an increased risk for renal cell carcinoma. Additional rigorous analytical studies are needed to confirm our results.

## INTRODUCTION

Approximately 337,860 cases of renal cell carcinoma (RCC) are diagnosed annually, and nearly 143,406 patients die from this cancer each year worldwide [1]. RCC is the third most common genitourinary malignancy. Moreover, both the incidence and mortality rates of RCC have steadily increased over the past several years [2]. The etiology of RCC is complex and multifactorial, and it involves multiple environmental and genetic factors [3,4]. Although an increasing number of studies have been performed on the etiology of RCC, the real causes of this cancer remain unclear. Previous studies have shown that many environmental factors such as cigarette smoking, alcohol drinking, occupational exposure to chemicals, hypertension and low frequencies of physical activity increase the risk of RCC [5–7]. Although many people are exposed to these risk factors during their lifetime, only a few people develop RCC. This finding suggests that genetic susceptibility plays a critical role in the etiology of this disease [8, 9].

Vascular endothelial growth factor (VEGF) is an important pro-angiogenic growth factor, and it is one of the most potent endothelial cell mitogens [10, 11]. VEGF plays a critical role in regulating the egress of the plasma proteins and cells that directly and indirectly stimulate angiogenesis [12]. Some research has indicated that the expression of VEGF affects tumor growth and metastasis, whereas the inhibition of *VEGF* signaling suppresses both tumor-induced angiogenesis and tumor growth [13]. The *VEGF* gene is located at chromosome 6p21.3 and consists of 8 exons. At least 30 single nucleotide polymorphisms (SNPs) exist in this gene [14] and some experimental studies have shown that certain SNPs can affect gene expression and change gene function [15].

Recently, numerous studies have been performed to evaluate the association between *VEGF* polymorphisms and RCC risk in diverse populations; however, the results of these studies conflict. To examine the association between *VEGF* polymorphisms and RCC risk, we performed a meta-analysis of all eligible published data up to June 5, 2016.

## RESULTS

### Study characteristics

**Figure 1:**
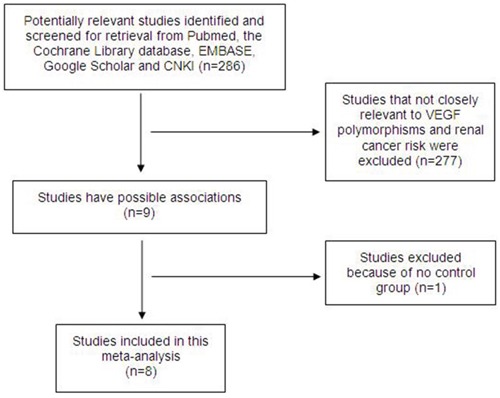
Flow diagram of the study selection

We performed a literature search, and 286 potentially relevant publications were identified. After screening the title and abstract of each study, 277 studies were excluded because they did not involve both *VEGF* polymorphisms and RCC risk. After the subsequent data extraction, one study was excluded because it lacked controls [16]. Finally, we obtained 8 relevant articles [17–24] that examined the association between *VEGF* polymorphisms and RCC risk (Figure 1); the data extracted from the articles are summarized in Table 1 . All of the included studies were evaluated using the Newcastle-Ottawa Scale (NOS) and were of high quality (Table 2). Of the 8 studies, 6 focused on the +936C/T polymorphism (rs3025039), 5 discussed −2578C/A (rs699947), 3 discussed +1612G/A (rs10434), -460T/C (rs833061) and −634G/C (rs2010963), and 2 studies examined both -405G/C (rs2010963) and -1154G/A (rs1570360). All of the included articles (excluding Shen et al.[20] and Lu et al. [21]) were case control studies, and their genotypic distributions across the controls followed Hardy-Weinberg Equilibrium (HWE).

**Table 1:** Characteristics of eligible studies in the meta-analysis of *VEGF* polymorphisms and RCC risk

Author	Year	Quality scores	Ethnicity	Design	Cases total	CC	CT	TT	Controls total	CC	CT	TT	*P* HWE
**+936C/T (rs3025039)**													
Abe A[17]	2002	5	Asian	HB	145	97	41	7	145	90	52	3	0.146
Bruyère F[18]	2010	5	Caucasian	PB	47	29	17	1	196	141	53	2	0.218
Sáenz-López P[19]	2013	6	Caucasian	PB	215	156	57	2	280	200	73	7	0.912
Shen BL[20]	2015	5	Asian	HB	360	224	81	55	359	240	73	46	0.000
Lu GJ[21]	2015	5	Asian	HB	412	262	91	59	825	554	166	105	0.000
Xian W[22]	2015	5	Asian	HB	266	70	127	69	532	196	236	100	0.056
**−2578C/A (rs699947)**					**Cases total**	**CC**	**CA**	**AA**	**Controls total**	**CC**	**CA**	**AA**	
Ajaz S[23]	2011	5	Asian	NA	143	30	81	32	106	44	41	21	0.053
Sáenz-López P[19]	2013	6	Caucasian	PB	216	54	114	48	272	77	142	53	0.388
Shen BL[20]	2015	5	Asian	HB	360	150	149	61	360	178	141	41	0.111
Lu GJ[21]	2015	5	Asian	HB	412	171	174	67	824	397	332	95	0.047
Xian W[22]	2015	5	Asian	HB	266	99	119	48	532	243	225	64	0.287
**+1612G/A (rs10434)**					**Cases total**	**GG**	**GA**	**AA**	**Controls total**	**GG**	**GA**	**AA**	
Abe A[17]	2002	5	Asian	HB	145	113	31	1	145	109	33	3	0.788
Shen BL[18]	2015	5	Asian	HB	361	152	170	39	360	166	164	30	0.234
Lu GJ[21]	2015	5	Asian	HB	412	172	191	49	825	365	375	85	0.431
**-460T/C (rs833061)**					**Cases total**	**TT**	**TC**	**CC**	**Controls total**	**TT**	**TC**	**CC**	
Bruyère F[18]	2010	5	Caucasian	PB	49	19	29	1	202	47	109	46	0.260
Sáenz-López P[19]	2013	6	Caucasian	PB	216	56	111	49	273	77	138	58	0.793
Lu GJ[21]	2015	5	Asian	HB	412	228	93	91	824	513	168	143	0.000
**−634G/C (rs2010963)**					**Cases total**	**GG**	**GC**	**CC**	**Controls total**	**GG**	**C**	**CC**	
Shen BL[20]	2015	5	Asian	HB	360	121	170	69	360	134	163	63	0.273
Lu GJ[21]	2015	5	Asian	HB	412	139	194	79	824	299	377	148	0.127
Xian W[22]	2015	5	Asian	HB	266	30	132	104	532	49	256	227	0.053
**-405G/C (rs2010963)**					**Cases total**	**GG**	**GC**	**CC**	**Controls total**	**GG**	**GC**	**CC**	
Bruyère F[18]	2010	5	Caucasian	PB	48	15	25	8	198	86	92	20	0.522
Sáenz-López P[19]	2013	6	Caucasian	PB	214	101	93	20	279	129	118	32	0.528
**-1154G/A (rs1570360)**					**Cases total**	**GG**	**GA**	**AA**	**Controls total**	**GG**	**GA**	**AA**	
Ricketts C[24]	2009	6	Caucasian	PB	324	134	143	47	314	146	130	38	0.281
Bruyère F[18]	2010	5	Caucasian	PB	49	27	17	5	202	94	83	25	0.322

HB, hospital-based controls; HWE, Hardy-Weinberg equilibrium.

**Table 2:** Quality assessment based on the Newcastle-Ottawa Scale of studies included in this meta-analysis^a^

Author	Year	Adequate definition of case	Representativeness of cases	Selection of control	Definition of control	Control for important factor or additional factor^b^	Exposure assessment	Same method of ascertainment for cases and controls	Nonresponse rate	Total quality scores
Abe A[17]	2002	★	★		★	★		★		5
Bruyère F[18]	2010	★	★	★	★			★		5
Sáenz-López P[19]	2013	★	★	★	★	★		★		6
Shen BL[20]	2015	★	★		★	★		★		5
Lu GJ[21]	2015	★	★		★	★		★		5
Xian W[22]	2015	★	★		★	★		★		5
Ajaz S[23]	2011	★	★		★	★		★		5
Ricketts C[24]	2009	★	★	★	★	★		★		6

a A study can be awarded a maximum of one star for each numbered item except for the item Control for important factor or additional factor.

b A maximum of two stars can be awarded for Control for important factor or additional factor.

### +936C/T (rs3025039)

**Figure 2:**
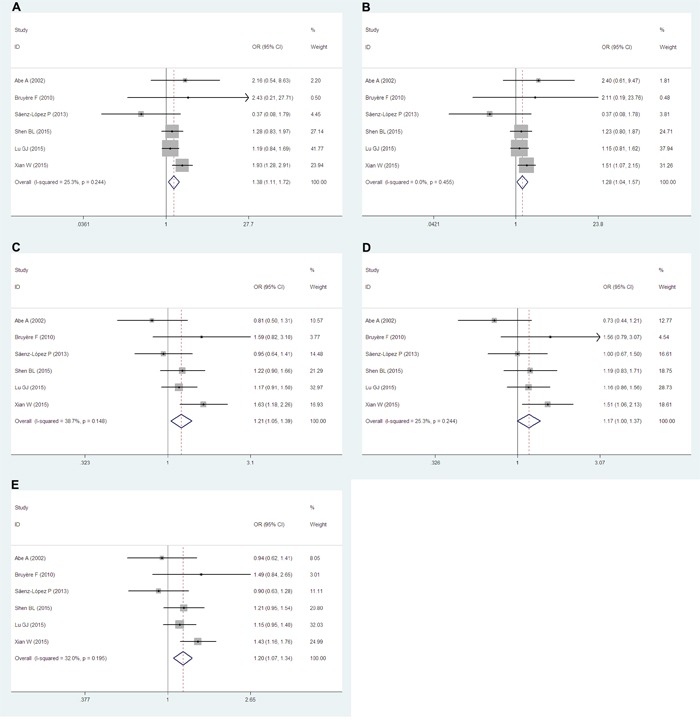
Forest plots of the +936C/T (rs3025039) polymorphism and RCC risk The squares and horizontal lines correspond to the study-specific ORs and 95% CIs. The areas of the squares reflect the study-specific weights (which was the inverse of the variance). The diamonds represent the pooled ORs and 95% CIs.

Six studies [17–22] including 1,445 cases and 2,337 controls examining the +936C/T (rs3025039) polymorphism were pooled. Overall, significant increased cancer risks were observed in most genetic models and alleles (TT vs. CC: odds ratio [OR]=1.38, 95% confidence intervals [CIs]=1.11-1.72, *P*=0.004, *I^2^*=25.3, Figure 2A; TT vs. CT+CC: OR=1.28, 95% CIs=1.04-1.57, *P*=0.019, *I^2^*=0.0, Figure 2B; TT+CT vs. CC: OR=1.21, 95% CIs=1.05-1.39, *P*=0.010, *I^2^*=38.7, Figure 2C; T vs. C: OR=1.20, 95% CIs=1.07-1.34, *P*=0.001, *I^2^*=32.0, Figure 2E) except CT vs. CC (OR=1.17, 95% CIs=1.00-1.37, *P*=0.056, *I^2^*=25.3, Figure 2D).

### −2578C/A (rs699947)

**Figure 3:**
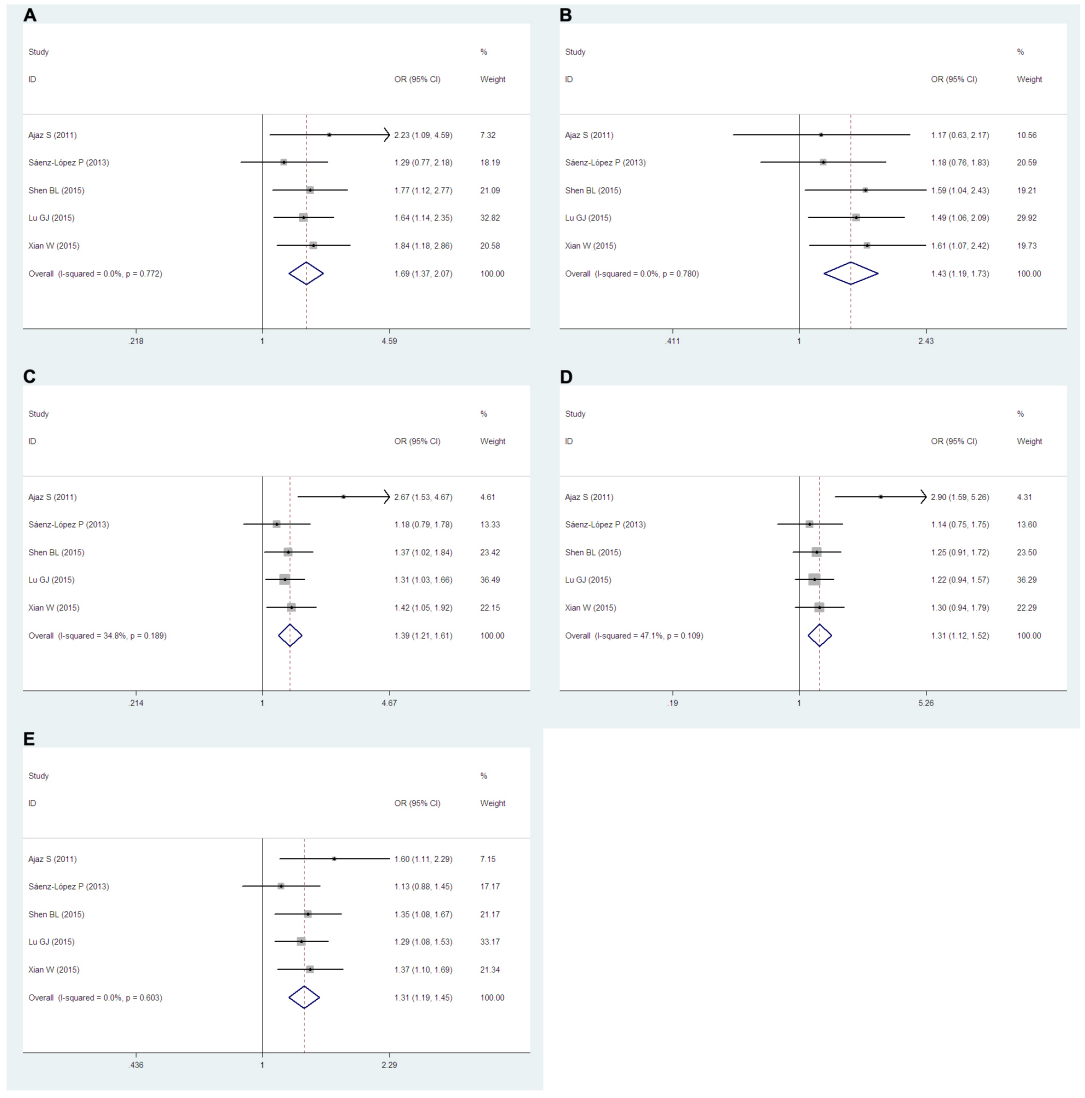
Forest plots of the −2578C/A (rs699947) polymorphism and RCC risk The squares and horizontal lines correspond to the study-specific ORs and 95% CIs. The areas of the squares reflect the study-specific weights (which were the inverse of the variance). Diamonds represent the pooled ORs and 95% CIs.

Five articles [19–25] including 1,397 cases and 2,094 controls examined the relationship between the −2578C/A (rs699947) polymorphism and RCC risk. Remarkably, significant associations were found in all genetic models (AA vs. CC: OR=1.69, 95% CIs=1.37-2.07, *P*=0.000, *I^2^*=0.0, Figure 3A; AA vs. CA+CC: OR=1.43, 95% CIs=1.19-1.73, *P*=0.000, *I^2^*=0.0, Figure 3B; AA+CA vs. CC: OR=1.39, 95% CIs=1.21-1.61, *P*=0.000, *I^2^*=34.8, Figure 3C; CA vs. CC: OR=1.31, 95% CIs=1.12-1.52, *P*=0.001, *I^2^*=47.1, Figure 3D), and also the A vs. C allele (OR=1.31, 95% CIs=1.19-1.45, *P*=0.000, *I^2^*=0.0, Figure 3E).

### +1612G/A (rs10434), -460T/C (rs833061) and −634G/C (rs2010963)

Three studies discussed the +1612G/A (rs10434) [17, 20, 21], -460T/C (rs833061) [18, 19, 21] and −634G/C (rs2010963) [20–22] polymorphisms. The numbers of participants in these studies were 918, 677 and 1,038 cases and 1,330, 1,299 and 1,716 controls, respectively. Unfortunately, no significant associations were found between RCC risks and in any genetic model or allele of these three polymorphisms.

### -405G/C (rs2010963) and -1154G/A (rs1570360)

We also investigated the -405G/C (rs2010963) [18, 19] and -1154G/A (rs1570360) [18, 24] polymorphisms, both of which were examined in two studies including 262 and 373 cases and 477 and 516 controls, respectively. However, we did not identify any association between RCC risk and either the -405G/C (rs2010963) or -1154G/A (rs1570360) polymorphism.

### Sensitivity analyses

Hardy-Weinberg disequilibrium was observed in two studies (Shen et al.[20] and Lu et al. [21]). For +936C/T (rs3025039) polymorphism, our sensitivity analyses results indicated that exclusion of the aforementioned studies did not change the results for all the genetic models and allele (data not shown). In addition, for −2578C/A (rs699947) polymorphism, the sensitivity analyses results for all the genetic models and allele did not change either when excluding the study of Lu et al. [21] (data not shown).

### Publication bias

Except for the -405G/C (rs2010963) and -1154G/A (rs1570360) polymorphisms, we used both funnel plots and Egger's test to assess the publication bias of each genetic model and allele. Our results did not show a publication bias for most of the genetic models and alleles (Supplementary Figure 1-2 showed the funnel plots of +936C/T and −2578C/A polymorphisms, respectively), except regarding CC vs. CT+TT of the -460T/C (rs833061) polymorphism (*P*=0.038).

## DISCUSSION

VEGF, a growth factor that regulates angiogenesis and is involved in promoting endothelial cell proliferation [25]. VEGF protein likely plays an important role in the development and progression of cancer. Researchers have found that the expression of *VEGF* is significantly related to tumor stage, tumor size, and nuclear grade in patients with clear cell RCC [26]. In addition, the overexpression of *VEGF* has been detected in the vast majority of RCC tissues [27]. Currently, *VEGF* inhibition is a therapy for RCC [28]. However, the *VEGF* gene is highly polymorphic [29] and several functional SNPs in the *VEGF* gene alter the expression of the VEGF protein, thereby affecting tumor growth and progression. Recent studies have investigated the association between SNPs in the *VEGF* gene and the risk of RCC. However, these results are controversial. Thus, we conducted this meta-analysis to discuss the relationship between *VEGF* polymorphisms and RCC risk.

Zhang et al. [30] previously performed a meta-analysis that observed the association between *VEGF* polymorphisms and RCC risk. However, the author only reviewed 5 studies. In contrast, our meta-analysis included 8 relevant published studies. Moreover, our meta-analysis included many more cases and controls than the prior meta-analysis. In addition, we evaluated the quality of studies using the NOS. All of the included studies met high-quality standards, whereas the prior meta-analysis did not conduct any quality assessment. Thus, our meta-analysis is a more convincing and detailed evaluation compared with the prior study. Overall, we found that significant associations exist between *VEGF* polymorphisms and RCC risk (all of our results are summarized in Table 3). Specifically, most genetic models and alleles found high risks of RCC regarding the +936C/T (rs3025039) polymorphism. To the best of our knowledge, our study is the first meta-analysis to report that the +936C/T (rs3025039) polymorphism of *VEGF* can increase the risk of RCC. The +936C/T (rs3025039) polymorphism is located in the 3′-UTR and likely associated with obviously increased serum VEGF levels [31], which are related to tumor stage, tumor size, and nuclear grade. Interestingly, according to the results of Krippl P [32], the carriers of a +936 T allele had significant decreased risks of breast cancer and lower serum VEGF levels, which is opposite with our results. The reason of this discrepancy may be the tumor heterogeneity. Tumor heterogeneity is complex in many levels, including interdisease, intertumor, intratumor and tumor-microenvironment heterogeneity, etc. [33]. Furthermore, significant RCC risks were found in all genetic models and alleles of the -2578C/A (rs699947) polymorphism, whereas the prior meta-analysis only found increased RCC risks for the AA vs. CC genetic model and the A vs. C allele. Currently, several studies have reported that the -2578C/A (rs699947) polymorphism in the promoter region plays an influential role regarding plasma VEGF levels [34, 35]. However, no significant associations were found between RCC risk and the +1612G/A (rs10434), -460T/C (rs833061), −634G/C (rs2010963), -405G/C (rs2010963) or -1154G/A (rs1570360) polymorphisms. All of the characteristics and results of the present study were compared with the former meta-analysis and summarized in Table 4.

**Table 3:** Summary of meta-analysis of *VEGF* polymorphisms and RCC risk

Polymorphism	No. of studies	No. of cases	No. of controls	Contrast	OR (95% CI)	Statistical method	I^2^%	*P*-value
+936C/T	6	1,445	2,337	TT vs. CC	1.38(1.11-1.72)	Fixed	25.3	0.004
(rs3025039)				TT vs. CT+CC	1.28(1.04-1.57)	Fixed	0.0	0.019
				TT+CT vs. CC	1.21(1.05-1.39)	Fixed	38.7	0.010
				CT vs. CC	1.17(1.00-1.37)	Fixed	25.3	0.056
				T vs. C	1.20(1.07-1.34)	Fixed	32.0	0.001
−2578C/A	5	1,397	2,094	AA vs. CC	1.69(1.37-2.07)	Fixed	0.0	0.000
(rs699947)				AA vs. CA+CC	1.43(1.19-1.73)	Fixed	0.0	0.000
				AA+CA vs. CC	1.39(1.21-1.61)	Fixed	34.8	0.000
				CA vs. CC	1.31(1.12-1.52)	Fixed	47.1	0.001
				A vs. C	1.31(1.19-1.45)	Fixed	0.0	0.000
+1612G/A	3	918	1,330	AA vs. GG	1.25(0.92-1.71)	Fixed	0.0	0.159
(rs10434)				AA vs. GA+GG	1.20(0.89-1.61)	Fixed	0.0	0.234
				AA+GA vs. GG	1.10(0.92-1.31)	Fixed	0.0	0.280
				GA vs. GG	1.08(0.90-1.30)	Fixed	0.0	0.423
				A vs. G	1.10(0.96-1.25)	Fixed	0.0	0.178
−460T/C	3	677	1,299	CC vs. TT	0.88(0.38-2.01)	Random	80.6	0.758
(rs833061)				CC vs. TC+TT	0.93(0.47-1.84)	Random	77.9	0.830
				CC+TC vs. TT	0.98(0.61-1.58)	Random	75.5	0.928
				TC vs. TT	1.12(0.89-1.41)	Fixed	31.0	0.343
				C vs. T	0.92(0.58-1.46)	Random	87.9	0.720
−634G/C	3	1,038	1,716	CC vs. GG	1.07(0.84-1.35)	Fixed	16.4	0.581
(rs2010963)				CC vs. GC+GG	1.00(0.83-1.20)	Fixed	0.0	1.000
				CC+GC vs. GG	1.09(0.91-1.30)	Fixed	0.0	0.370
				GC vs. GG	1.08(0.89-1.31)	Fixed	0.0	0.429
				C vs. G	1.03(0.92-1.16)	Fixed	27.7	0.571
−405G/C	2	262	477	CC vs. GG	1.26(0.45-3.51)	Random	68.4	0.661
(rs2010963)				CC vs. GC+GG	1.11(0.51-2.41)	Random	54.5	0.796
				CC+GC vs. GG	1.18(0.70-2.01)	Random	52.5	0.536
				GC vs. GG	1.11(0.80-1.55)	Fixed	13.0	0.532
				C vs. G	1.14(0.72-1.79)	Random	67.0	0.584
−1154G/A	2	373	516	AA vs. GG	1.19(0.77-1.84)	Fixed	19.9	0.435
(rs1570360)				AA vs. GA+GG	1.14(0.76-1.73)	Fixed	0.0	0.528
				AA+GA vs. GG	1.00(0.59-1.69)	Random	58.1	0.994
				GA vs. GG	1.08(0.80-1.46)	Fixed	45.3	0.611
				A vs. G	1.01(0.68-1.51)	Random	57.1	0.948

**Table 4:** Characteristics and results of the present study compared with the previous meta-analysis

Polymorphism	Contrast	No. of studies		No. of cases		No. of controls		Overall results	
		previous	present	previous	present	previous	present	previous	present
+936C/T	TT vs. CC	3	6	407	1,445	621	2,337	–	+
(rs3025039)	TT vs. CT+CC							–	+
	TT+CT vs. CC							–	+
	CT vs. CC							–	–
	T vs. C							–	+
−2578C/A	AA vs. CC	2	5	359	1,397	378	2,094	+	+
(rs699947)	AA vs. CA+CC							–	+
	AA+CA vs. CC							–	+
	CA vs. CC							–	+
	A vs. C							+	+
+1612G/A	AA vs. GG	NA	3	NA	918	NA	1,330	NA	–
(rs10434)	AA vs. GA+GG							NA	–
	AA+GA vs. GG							NA	–
	GA vs. GG							NA	–
	A vs. G							NA	–
−460T/C	CC vs. TT	2	3	265	677	475	1,299	–	–
(rs833061)	CC vs. TC+TT							–	–
	CC+TC vs. TT							–	–
	TC vs. TT							–	–
	C vs. T							–	–
−634G/C	CC vs. GG	NA	3	NA	1,038	NA	1,716	NA	–
(rs2010963)	CC vs. GC+GG							NA	–
	CC+GC vs. GG							NA	–
	GC vs. GG							NA	–
	C vs. G							NA	–
−405G/C	CC vs. GG	2	2	262	262	477	477	–	–
(rs2010963)	CC vs. GC+GG							–	–
	CC+GC vs. GG							–	–
	GC vs. GG							–	–
	C vs. G							–	–
−1154G/A	AA vs. GG	2	2	373	373	516	516	–	–
(rs1570360)	AA vs. GA+GG							–	–
	AA+GA vs. GG							–	–
	GA vs. GG							–	–
	A vs. G							–	–

Certain limitations of this meta-analysis should be acknowledged. First, because our study only considered published articles, a publication bias might exist. However, the publication bias was only found for the CC vs. CT+TT of -460T/C (rs833061) polymorphism. The statistical results of the funnel plot and Egger's test support this finding. Second, the heterogeneities among certain genetic models and alleles were significant. The reasons underlying these heterogeneities included the source of the controls, the study design and differences in genetic backgrounds. Third, the control sample of two articles were in Hardy-Weinberg disequilibrium, however, all the results of +936C/T (rs3025039) and -2578C/A (rs699947) polymorphisms did not change significantly after sensitivity analyses. Fourth, as the most of the cases of +936C/T and -2578C/A polymorphisms were from Asians, so our results of these two SNPs may not represent Caucasians. Finally, because of the use of unadjusted data, potential confounds such as age, sex and residence might also have affected the effect estimates. Thus, a more precise and large scale evaluation based on adjusted data is needed.

In summary, our meta-analysis suggests that the +936C/T (rs3025039) and -2578C/A (rs699947) polymorphisms of *VEGF* are associated with increased risks for RCC. However, no significant RCC risks were obtained regarding the +1612G/A (rs10434), -460T/C (rs833061), -634G/C (rs2010963), -405G/C (rs2010963) or -1154G/A (rs1570360) polymorphisms. To the best of our knowledge, this meta-analysis is the first to report that the +936C/T (rs3025039) polymorphism can increase the risk of RCC. Larger and more rigorous analytical studies are required to confirm our results and evaluate the gene-environment interactions with regard to RCC risk.

## MATERIALS AND METHODS

### Search strategy and selection criteria

According to the Preferred Reporting Items for Systematic Reviews and Meta-Analyses (PRISMA), we performed an electronic systematic search of PubMed, the Cochrane Library database, EMBASE, Google Scholar and the China National Knowledge Infrastructure (CNKI) without any restriction on language up to June 5, 2016. The combinations of keywords used were “renal cancer” or “renal carcinoma”; “polymorphism” or “variant”; and “vascular endothelial growth factor” or “VEGF.” In addition, the reference lists of the papers retrieved and recent reviews were also examined. We included all studies that (1) evaluated the association between *VEGF* polymorphisms and the risk of RCC in humans; (2) used a case control design; (3) confirmed RCC using the accepted diagnostic criteria; (4) reported sufficient published data, including ORs and their 95% CIs, or the number of events for the purposes of calculation. The exclusion criteria were (1) a lack of sufficient data to calculate ORs with corresponding 95% CIs; and (2) overlapping cases or controls. Only the most recent or the largest research study was included in the case of overlap.

### Data extraction

Two investigators (GMC and DWJ) extracted the raw data independently based on the inclusion and exclusion criteria. The following information was extracted from all of the enrolled studies (see Table 1): the surname of the first author, date of publication, participant ethnicity, quality scores, sources of controls, number of cases and controls and the HWE *P-*value. All disagreements were resolved via discussion.

### Quality assessment

Two authors (GMC and SZR) assessed the study quality using the NOS [36] which evaluates methodological quality using a star rating system. Nine stars was defined as a full score; 5 to 9 stars was considered as being of high methodological quality; and 0 to 4 stars was considered as being of poor quality [37]. The quality of all the included studies is listed in Table 2. For conflicting NOS scores, an agreement was reached via a comprehensive reassessment, and only high-quality studies were included in our meta-analysis.

### Statistical analysis

The relationship between *VEGF* polymorphisms and the risk of RCC was evaluated via pooled ORs with 95% CIs. The significance of the pooled ORs was tested using the *Z*-test, and a (two-tailed) *P*-value of <0.05 was regarded as significant. The HWE was calculated in the control groups using the chi-square test, and *P*<0.05 signified a departure from HWE. Between-study heterogeneity was calculated using the *I^2^* test. If the heterogeneity was significant (*I^2^*>50%) [38], then a random-effects model was used (the DerSimonian and Laird method) [39]; otherwise, the fixed-effect model (the Mantel-Haenszel method) [40] was applied. To assess the stability of the results, sensitivity analyses were conducted to evaluate the impact of the studies, especially which not in HWE. Because publication bias is always a concern for meta-analyses, funnel plots and Egger's test were both used to examine publication bias (*P*<0.05 was considered as significant publication bias) [41]. All statistical analyses were performed using STATA statistical software (Version 12.0; Stata Corporation, College Station, TX, USA).

## SUPPLEMENTARY MATERIALS FIGURES


