# Supplementary material for: Vascular endothelial growth factor gene polymorphisms and the risk of renal cell carcinoma: Evidence from eight case-control studies
Source: Oncotarget. 2016 Dec 27;8(5):8447–58. doi: 10.18632/oncotarget.14263 (PMC5352413; doi:10.18632/oncotarget.14263)
Supplement: Supplementary file 1 [file oncotarget-08-8447-s001.pdf]

Vascular endothelial growth factor gene polymorphisms and the risk of renal cell carcinoma: Evidence from eight case-control studies

SUPPLEMENTARY FIGURES

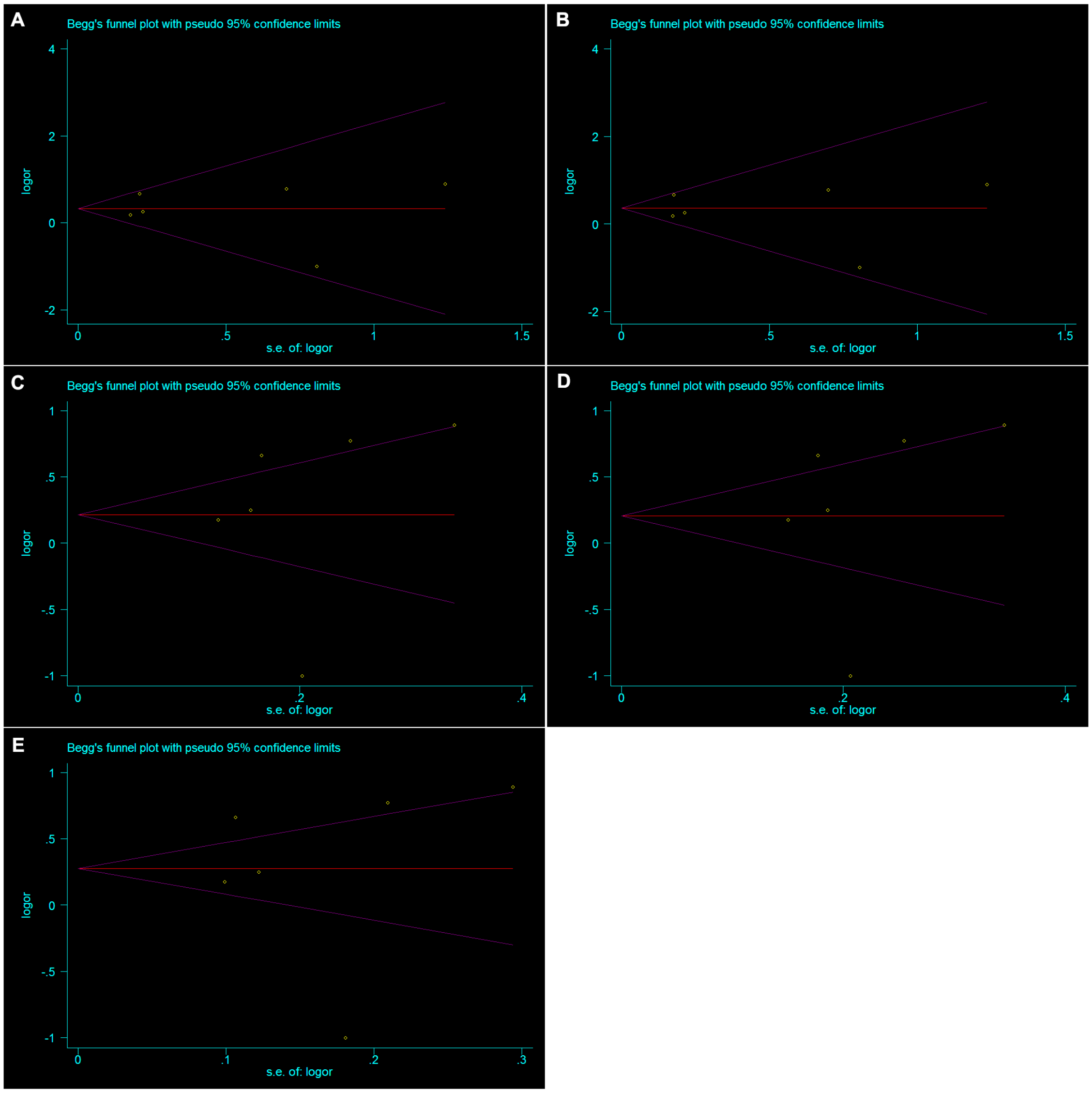

Supplementary Figure 1: Funnel plot of +936C/T (rs3025039) polymorphism (A for TT vs. CC; B for TT vs. CT+CC; C for TT+CT vs. CC; D for CT vs. CC and E for T vs. C).

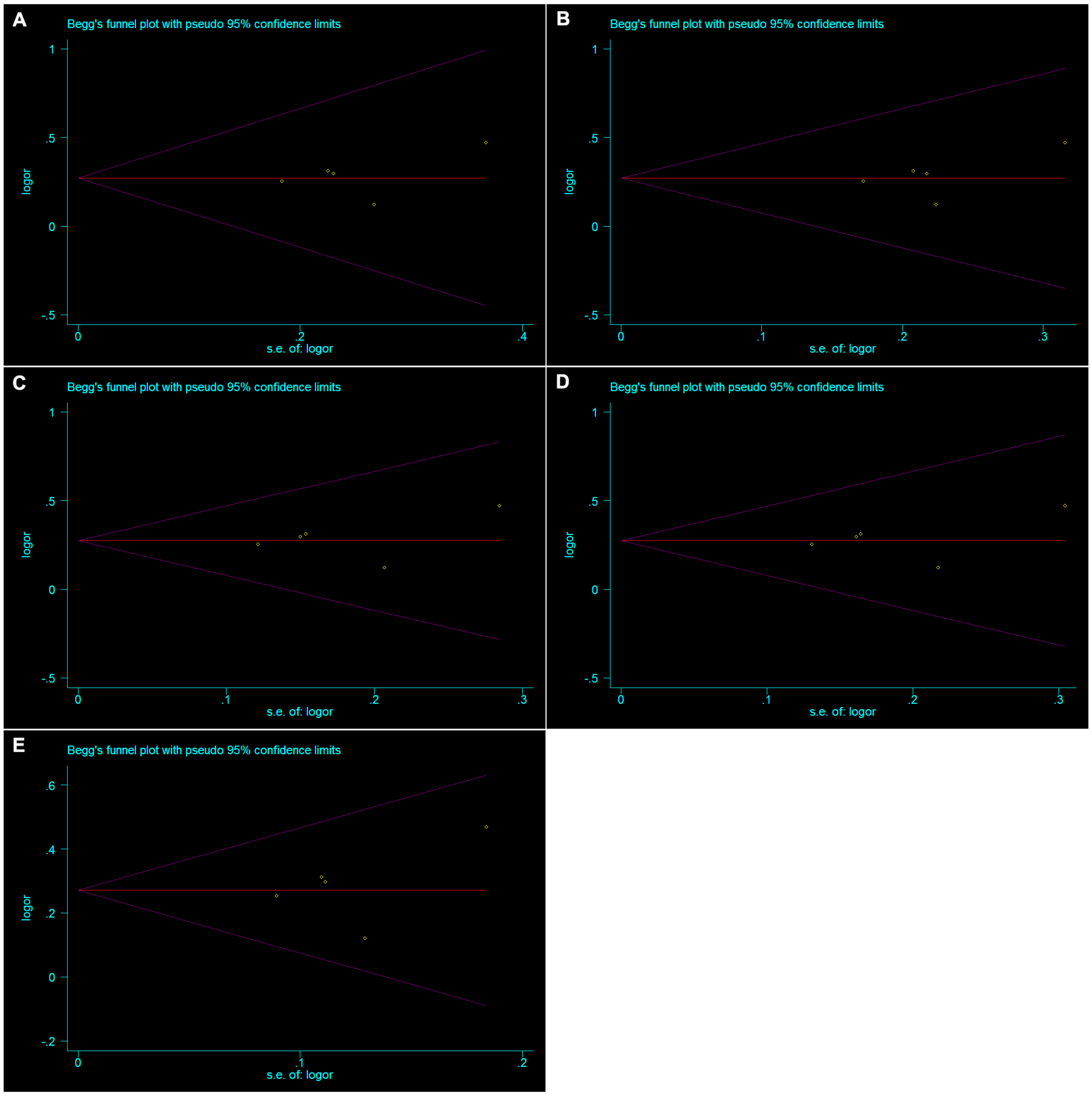

Supplementary Figure 2: Funnel plot of −2578C/A (rs699947) polymorphism (A for AA vs. CC; B for AA vs. CA+CC; C for AA+CA vs. CC; D for CA vs. CC and E for A vs. C).
